# Commentary: Serum hepatitis B virus large and medium surface proteins as novel tools for predicting HBsAg clearance

**DOI:** 10.3389/fimmu.2022.1081730

**Published:** 2022-12-02

**Authors:** Maria Pfefferkorn, Florian van Bömmel

**Affiliations:** Division of Hepatology, Department of Medicine II, Leipzig University Medical Center, Leipzig, Germany

**Keywords:** HBsAg composition, HBV, LHBs, Hepatitis B surface antigen, MHBS

## Introduction

With great interest, we read the recently published article from Lin X and colleagues describing the association of the different components of hepatitis B surface antigen (HBsAg) with the outcome of antiviral treatment. (1) In their manuscript, the authors report that the levels of large (LHBs) and middle (MHBs) components of HBsAg can predict HBsAg clearance following PEG-IFN therapy in inactive HBsAg carriers, and the predictive value for LHBs and MHBs is higher than that of total HBsAg levels. Although the components of HBsAg have been the subject of research for some time, they have only recently been investigated for their potential value as biomarkers of hepatitis B virus (HBV) infections that can provide insights beyond the established markers HBV DNA or HBsAg or HBeAg. In this context, we and others have previously reported that the proportion of the HBV surface proteins varies upon the different phases of acute and chronic HBV infection and upon the HBV genotype. (2, 3) We found high levels and proportions of MHBs significantly associated with a high replicative state of HBV infection. In contrast, low proportions of LHBs in patients were associated with a low-replicative HBeAg-negative chronic HBV infection, the so called “inactive carrier state” (IC). (2) More than two decades ago, other groups could already demonstrate the special pattern of MHBs and LHBs loss prior to HBsAg loss in patients with acute or chronic hepatitis B. (4, 5) Moreover, our group found the proportions of MHBs and LHBs to decrease prior to HBsAg loss in patients with chronic hepatitis B (CHB) during nucleos(t)ide analogue treatment or PEG-IFN based treatment, suggesting that these proteins might represent promising novel biomarker candidates to predict functional cure. (6) We are excited to learn that the findings from Lin and colleagues further strengthen those observations in a, however, different patient population which consisted of ICs that were treated with PEG-IFN. What is also similar to our findings is that a combination of baseline MHBs and LHBs levels as well as the decrease at treatment week 12 seemed to be stronger associated with subsequent functional cure than total HBsAg levels at baseline or week 12. (7, 8) We believe that there is now enough evidence for the high potential of HBsAg components to develop them further as treatment markers and to validate them in clinical trials.

## Detailed validation and methodology needed

In order to establish the quantification of HBsAg proteins as diagnostical tool, detailed methodology needs to be shared to replicate results on international basis. Therefore, the crucial step in the quantification seems to target LHBs and MHBs separately, since both proteins share together with SHBs the same C-terminal gene sequence, thus amino acid sequence. (9) Consequently, they can only be individually quantified targeting either posttranslational modifications or the additional 109-118 aa sequence of LHBs. To tackle this problem, we as well as other groups used well-defined monoclonal antibodies to target either LHBs or MHBs. After characterizing the preS gene and its products in the 1990s, Gerlich and colleagues defined the binding epitopes and properties of those antibodies in different studies. (10–12) Others also used a mathematical approach to quantify all HBsAg proteins *via* antibody recognition in the different preS1/2 and S-domain and calculated the presumed proportion of MHBs and SHBs *via* substraction. (13)

Another challenge in quantifying the HBsAg proteins represents the usage of a consistent standard, in which the amount of LHBs and MHBs is determinated and approved. In the past, our group used the well-defined ID1 standard and assessed the amount of LHBs and MHBs *via* semi-quantitative western blot to provide a validated quantification system. (14) However, most studies lack information about the usage of a standard and thus comparable results are difficult to obtain. However, the detailed description of the quantification is fundamental to further investigate and validate the importance of the HBsAg proteins as prediction marker in HBV cure.

## Baseline cutoffs as future tool for the prediction of functional cure?

In our previous works, we have described LHBs and MHBs as a proportion of total HBsAg. Lin and colleagues demonstrate a different approach by using cutoff baseline quantities of LHBs and MHBs (in ng/mL). Comparing the quantities of the presented IC patients, levels of LHBs and MHBs are comparable to results prior published by our group (see **Table 1**). However, further validation is needed to use this cut-off approach, since the quantities of LHBs and MHBs differ regarding the state of replication and disease.

**Table 1:** Mean quantities and ratios of L, M and SHBs in different HBV patient groups and comparison of IC to other disease stages; prior published in (2).

	a) ICs	b) Acute infections	c) HBeAg-negative CHB	d) HBeAg-positive phase	e) HBV/HDV-co-infection	p-value			
						a) vs. b)	a) vs. c)	a) vs. d)	a) vs. e)
***LHBs*** (log_10_ ng/mL)*	1.9±0.5 (-1.9-2.92; 1 nd)	2.6±0.8 (-0.9.4-3.5)	2.5±0.6 (0.7-3.6)	3.1±0.6 (1.4-4.3)	2.9±0.7 (1.7-3.8)	0.0006	3.2x10^-7^	4.0x10^-8^	4.0x10^-5^
***MHBs*** (log_10_ ng/mL)*	1.8±0.6(0.8-2.9; 10 nd)	2.6±1.3 (-0.9-3.9; 1 nd)	2.1±0.8 (0.1-3.5; 1nd)	2.6±0.8 (0.8-4.4)	2.7±0.9 (2.1-4.9)	0.0003	0.0003	1.8x10^-10^	3.0x10^-4^
***SHBs*** (log_10_ ng/mL)*	3.1±1.1 (0.5-4.5)	3.5±1.3 (0.3-4.9)	3.6±0.5 (2.1-4.8)	4.1±0.7 (2.3-5.6)	3.9±0.7 (2.5-4.8)	0.1999	0.0777	2.8x10^-8^	0.0111
***total HBsAg*** (log_10_ ng/mL)*	3.1±1.1 (0.5-4.5)	3.6±1.2 (0.3-4.9)	3.7±0.6 (2.1-4.8)	4.2±0.7 (2.4-5.7)	4.0±0.6 (2.6-4.8)	0.1084	0.4809	7.6x10^-9^	0.0051
***LHBs*** (%)*	2.3±1.6 (0.0-7.5)	5.7±2.4 (1.9-11.3)	6.0±3.3 (0.5-22.0)	7.6±4.0 (2.0-20.8)	9.2±3.0 (5.8-14.5)	1.0x10^-5^	3.1x10^-12^	3.3x10^-18^	7.3x10^-10^
***MHBs*** (%)*	1.8±1.9 (0.0-7.7)	9.1±6.8 (0.0-24.6)	4.4±4.3 (0.0-22.0)	4.3±4.0 (0.1-24.2)	9.9±10.8 (0.4-37.3)	8.3x10^-4^	8.3x10^-4^	7.0x10^-5^	0.0015
***SHBs*** (%)*	95.9-2.6 (89.6-100.0)	85.2±7.8 (69.1-98.0)	89.6±5.8 (65.7-99.0)	88.1±6.7 (64.9-97.3)	80.9±12.9 48.1-93.7	4.1x10^-7^	4.1x10^-11^	7.3x10^-15^	8.3x10^-9^

*=mean±SD (range); nd, undetectable.

Using this approach to predict functional cure in patients with chronic hepatitis B, cut-off limits should be assessed in all stages of HBV infections. However, different quantification methods might lead to different levels of LHBs and MHBs, thus cut-off-based prediction might become difficult. To overcome this problem, proportions of the HBsAg proteins might be assessed and their value in treatment response prediction needs to be further investigated.

## Discussion

The quantification of the HBsAg proteins may be a promising tool to predict the achievement of functional cure during NA and PEG-IFN treatment as shown in previous studies HBsAg components might also play a role for patient selection for novel HBV treatments, which are currently under investigation and might be associated with side effects or high costs. However, the underlying cause for the association of HBsAg composition and achievement of functional cure still needs to be revealed. In addition, the HBsAg composition might be associated with HCC progression and recurrence, which underlines the need for further investigations (13, 15). However, as different methods in the quantification of the HBsAg proteins have been proposed, a validation of different assays is required, especially regarding a cut-off-based approached as it is proposed in the present work by Lin et al. Open questions remain regarding the origin an biological role of HBsAg components and their potential as a surrogate marker for cccDNA activity, a key player in the chronic HBV infection (16).

## Author contributions

Coordination, drafting and revising of the manuscript was done by both authors. All authors contributed to the article and approved the submitted version.

## Funding

This research was partially supported by Grant DLR 01ES0821 to FB from the German Ministry of Education and Research (BMBF).

## Conflict of interest

The authors declare that the research was conducted in the absence of any commercial or financial relationships that could be construed as a potential conflict of interest.

## Publisher’s note

All claims expressed in this article are solely those of the authors and do not necessarily represent those of their affiliated organizations, or those of the publisher, the editors and the reviewers. Any product that may be evaluated in this article, or claim that may be made by its manufacturer, is not guaranteed or endorsed by the publisher.
